# Historic tobacco legislation in Israel: a moment to celebrate

**DOI:** 10.1186/s13584-020-00384-3

**Published:** 2020-05-04

**Authors:** L. Rosen, S. Kislev, Y. Bar-Zeev, H. Levine

**Affiliations:** 1 Department of Health Promotion, School of Public Health, Sackler Faculty of Medicine, Tel Aviv University, POB 39040, 69978 Ramat Aviv, Israel; 2 The National Initiative to Eradicate Smoking (Smoke-Free Israel), Ramat Raziel, Israel; 3 Braun School of Public Health and Community Medicine, Hebrew University-Hadassah Faculty of Medicine, P.O Box 12272, Kiryat Hadassah, Ein Kerem, 91120 Jerusalem, Israel

**Keywords:** Tobacco control, Legislation, Israel, Advertising and marketing ban, Electronic cigarettes, Vaping, Nicotine

## Abstract

**Background:**

Israel was once a leader in tobacco control, but fell behind other countries, particularly during the past decade, as smoking rates stagnated.

**Text:**

Landmark tobacco control legislation, which banned advertising (with the exception of the print press) and limited marketing, was passed in Israel on Dec. 31^rst^, 2018. The changes occurred following years of attempts which culminated in successful last-minute efforts to promote the legislation just before the early disbanding of the 20th Knesset (Israeli Parliament). Regulations concerning marketing and advertising were substantially strengthened to address all tobacco, nicotine and smoking products. Digital media was included for the first time. Electronic cigarettes, which were previously largely unregulated, now fall under existing tobacco legislation. The changes overcame intense opposition from the tobacco lobby, and occurred despite the fact that the basic elements for prevention policy postulated by the Richmond model were not in place.

**Conclusions:**

This legislation represents an important and long-awaited change in Israeli tobacco control policy. Many deficiencies in existing tobacco control regulation were overcome, and some measures went beyond current international regulations. The cohesive partnership between legislators, public health organizations and professionals, advocacy groups, academia, and leading journalists was critical to this success. The progress was lauded by the World Health Organization with its highest award for tobacco control, which was presented to Smoke Free Israel. This case study provides important lessons for up-to-date tobacco control policy, in the age of rapid global changes in the tobacco, vaping and nicotine landscape.

## Background

Israel became an international leader in tobacco control with its 1983 passage of landmark national legislation restricting advertising of tobacco products (including a ban on radio and television advertising, at cinema screenings, billboards, educational facilities, and on public transportation), and banning smoking in public places (including cinemas and theatres, medical facilities, busses and taxis, elevators, and educational institutions). These regulations were passed two decades before the formation of the World Health Organization’s Framework Convention on Tobacco Control (FCTC), which Israel signed in 2003 and ratified in 2005. The Plan for the Reduction in Tobacco Use and Damage was passed by the government in 2011 [1]; that plan included numerous provisions, such as extensions to the law banning smoking in public places, and strengthening Israel’s advertising and marketing regulations. The additional restrictions on smoking in public places were passed by the Knesset and went into effect in 2012. However, the proposed amendment to the Law Restricting Advertising and Marketing of tobacco products, though submitted to the Knesset by the Ministry of Health, was withdrawn in 2014, following tobacco industry interference [2].

Through Dec. 31, 2018, Israel’s tobacco control policy was as follows:

1. Restrictions on advertising and marketing. After 1983, this law was extended to include mandatory, exchangeable health warnings; a prohibition on the use of signs or words such as “light” to indicate harm-reduced products; the inclusion of additional tobacco products, (including combustible tobacco products such as nargilla (hookah), and non-combustible products such as snus); a ban on sales to minors; and a ban on tobacco vending machines [2].
2. Bans on smoking in public places. This was extended over time to include, among other venues, bars and pubs (with exemptions for smoking areas), restaurants, sports venues, event halls, concerts, public events with more than 50 people, zoos, railway platforms and covered bus stops, parking lots, and swimming pools [3].
3. Taxation was first instituted in 1952 for the purpose of consumer product tax, not tobacco control; this was later expanded with the purpose of controlling tobacco use [2]. By 2013, the amount and type of taxation had increased to 80% of cigarette packs, 36% of roll-your-own (RYO) packages, 90% of cigars and 33% of nargila [3]. In 2018, Philip Morris’s heated tobacco product, IQOS, became subject to equal taxation as cigarettes [4].
4. Support for smoking cessation (free behavioural counseling, plus subsidization of medications for those who receive counseling) through the National Basket of Services. From 2010, prescription medications Varenicline and Bupropion SR were subsidized for smokers who attended free smoking cessation group behavioral counselling. In 2018, Nicotine Replacement Therapy (NRT) was added as an option for those who attended smoking cessation group behavioral counselling but were not able to receive subscription medications for medical reasons.
5. While tobacco products, including heated tobacco products, were included under the previous legislation, e-cigarettes were not regulated, with the exception of a ban on high-nicotine (> 20 mg) e-cigarette refills and pods.

In recent years, Israeli tobacco control policy fell behind that of other countries, while population smoking rates, which had been steadily decreasing since data on prevalence were first collected in the mid-1970’s, have been stagnant for some years at about 20% prevalence among adults [3]. The Israeli State Comptroller in 2018 criticized various Ministries and organizations regarding lacunae in tobacco control; in particular, it labeled as “extremely serious” the failure of the Ministry of Health to promote strengthening of the Law on Advertising and Marketing of Tobacco Products [5]. In this paper, we describe the changes incorporated into the 2018 amendments to the Advertising and Promotion law, and describe the elements which led to the successful passage of that legislation following years of stagnation.

## Methods

We report on the law banning advertisement and restricting marketing of tobacco products, which was passed into law by the Knesset on December 31, 2018. In order to describe the process, summarize the legislation, and put that legislation in an international context, we used governmental and legal documents, reports by the World Health Organization, the scientific literature, and media reports. In addition, the authors attended Knesset meetings as the legislation was progressing, had direct contacts with Knesset members and their aides, and made media appearances.

## Results

On Dec. 31, 2018, the Knesset (Israeli Parliament) passed historic tobacco control legislation regarding tobacco marketing and advertisement restrictions. This legislation, entitled “the Ban on Advertising and Restrictions on Marketing Law” represents the most far-reaching change in Israeli tobacco policy since the first national legislation was passed in 1983.

### Summary of regulations

Table 1 presents previous and new regulation progress in the context of the World Health Organization’s Framework Convention on Tobacco Control (FCTC) and MPOWER measures [6].

**Table 1:** Israel Legislation Progress in the Context of the World Health Organization’s FCTC and MPOWER measures

Prior to the 7th Amendment to the law on Restrictions on Advertising and Marketing of Tobacco Products	Following the Dec. 31, 2018 passage of the Ban on Advertising and Restrictions on Marketing of Tobacco and Smoking Products	Framework Convention of Tobacco Control Articles	MPOWER Score^a^
Addressed only tobacco products (combustible, smokeless and heated)	Includes both tobacco products (combustible, smokeless, and heated) and any smoking and/or vaping products	NA	NA
Specified restrictions on advertisement	Complete ban on advertisement	Article 13.4.e:	MPOWER^a^ - Enforce bans on advertising, promotion and sponsorship
	Except:	Undertake a comprehensive ban or, in the case of a Party that is not in a position to undertake a comprehensive ban due to its constitution or constitutional principles, restrict tobacco advertising, promotion and sponsorship on radio, television, print media and, as appropriate, other media, such as the internet …	
	1. Newspapers^b^		
	2. Specialised tobacco and/or alcohol shops		The new legislation did not improve MPOWER score compared to the previous legislation:
	3. Art objects		
	4. Direct written mailing to over 21 years old, with pre-authorization		
			Score 3: Ban on national TV, radio and print media as well as some (but not all) other forms of direct and/or indirect advertising
Ban on using human or animal figures, including cartoons, to market tobacco products	Expanding the ban to include also the use of fruit or any kind of plant figure (refers to allowed ads only and packaging)	Article 13.4.a:	
		Prohibit all forms of tobacco advertising, promotion and sponsorship that promote a tobacco product by any means that are false, misleading or deceptive or likely to create an erroneous impression about its characteristics, health effects, hazards or emissions;	
General unspecified restriction on using indirect advertisement for tobacco products	1.Specifies that no use can be made of tobacco/smoking products brand name/nickname/symbols and/or resemblance of these to promote other products	Article 16.1.c:	
		Prohibiting the manufacture and sale of sweets, snacks, toys or any other objects in the form of tobacco products which appeal to minors	
	2. Ban on sales of any candy or toys that resemble cigarettes		
Warning area on package 30% for tobacco products, includes rotating print warnings	1. For tobacco products – warning area increased to 65% of package, continues to include rotating print warnings	Article 11:	MPOWER - Warning labels on tobacco packaging
		Packaging and labelling of tobacco products	
		Article 13.4.b:	
	2. For vaping products – warning area 30%, warning, will read “this product is highly addictive and harmful to your health”		
		Require that health or other appropriate warnings or messages accompany all tobacco advertising and, as appropriate, promotion and sponsorship	The new legislation improved the MPOWER score from 3 to 4
	3. For smoking products (without tobacco, such as herbal) - warning area 30%, warning, will read “smoking causes morbidity and premature death”		
			Score 4: Medium size warnings with all appropriate characteristics OR Large warnings missing some appropriate characteristics
Does not include any restrictions on point-of-sale placement	All products will not be visible to the consumers	Article 16.1.b:	NA - Additional measures not included in the MPOWER
	Except:	Banning the sale of tobacco products in any manner by which they are directly accessible, such as store shelves	
	1. Specialised tobacco and/or alcohol shops (as long as not visible from outside the store)		
	2. Duty-free shops (as long as will not be visible from the outside or from other parts of the shop)		
	3. Dedicated online shops (allowed to only include specific details of the product without pictures)		
Did not include any regulation on contents of products	Restricts the nicotine content in vaping products to 20 mg/ml maximum	Article 9	
		Regulation of the contents of tobacco products	
Did not include any reference to vaping products	Vaping products must be sold in child-proof packaging	NA	
Restricting sales to underage minors (< 18 years old) of tobacco products	Extending this restriction to include also all vaping products and any herbal smoking products	Article 16.1:	
		Each Party shall adopt and implement effective legislative … .to prohibit the sales of tobacco products to persons under the age …	
Did not include plain packaging	All tobacco/smoking/vaping products will use plain packaging utilizing the colour Pantone 448 C (except cigars and pipe tobacco)	Article 11:	
		Packaging and labelling of tobacco products	
Did not include package inserts	All tobacco/smoking/vaping products will include a package insert with information on the harms of using these products and referral pathways to cessation support	Article 13.4.b:	
		Require that health or other appropriate warnings or messages accompany all tobacco advertising and, as appropriate, promotion and sponsorship	
Did not include details regarding product content, emission or toxicological data	Requires all manufacturers/importers to disclose once a year all information regarding the content of their products, levels of emissions, and any toxicological data	Article 10:	
		Regulation of tobacco product disclosures	

^*^Note change in the name of the bill

^a^MPOWER score according to the WHO Technical note 1: Evaluation of existing policies and compliance, range from 0 to 4 https://www.who.int/tobacco/global_report/en/

^b^Newspapers were exempt from the complete advertisement ban, but the legislation does include a few expansions to previous restrictions: 1) For each ad, a counter ad by the MOH will be published in the same newspaper; 2) Only one ad per each printed edition; 3) Warning area extended from 5 to 30% of the ad

The new law includes the following key elements:

1. The title of the law was revised. Previously, it was called Restrictions on Advertising and Marketing of Tobacco Products. This proposal was called “the 7^th^ Amendment to the law on Restrictions on Advertising and Marketing of Tobacco Products”. The name of the law passed was revised to “Ban on Advertising and Restrictions on Marketing of Tobacco and Smoking Products”.
2. The definition of products which are covered under the law was expanded. Previously, the law referred to “tobacco products.” The revision places the following products under the law: tobacco products (including IQOS and other heated tobacco products), smoking products which are based on herbs, products used such as papers for roll-your-own cigarettes, and electronic cigarettes, whether the refill liquid includes or does not include nicotine. With this redefinition, products, such as e-cigarettes, which were not previously covered by the advertising and marketing law or the law regarding bans on smoking in public places, became subject to all sections of both these laws. Of note is that e-cigarettes can no longer be sold to minors, just as other tobacco products cannot be sold to minors. Also, because the definition of smoking in the law banning smoking in public places is defined by the Advertising law, this expanded definition now applies to the laws banning smoking in public places as well.
3. Specific regulation on e-cigarettes include a limitation on nicotine content, and the requirement for child-proof caps on liquids (From 03/2019).
4. Advertisement is banned in all forms, including the internet and points-of-sale, with the exception of print press aimed at adults. (Since 2006, there has been a complete ban on tobacco product advertisement aimed at minors.) Ads in the print press must be accompanied by same-size, tobacco-industry-funded, Ministry-of-Health written anti-tobacco ads (From 03/2019). Direct advertising by text messaging and email delivered to recipients aged over 21 following written signed consent is permitted.
5. Use of pictures of animals, fruits, or flowers on packages will be banned (From 06/2019).
6. Displays of all included products will be banned, with the exception of in smoking or alcohol outlets (From 01/2020).
7. Plain packaging will be mandatory (color pantone 448c (as in Australia)). Text warnings will cover 65% of the package area for cigarettes, smoke-less, and heated tobacco products, and 30% of the package area for e-cigarettes (From 01/2020).
8. Package inserts with warnings by the Ministry of Health will be mandatory, and based on specific instructions from the Minister of Health.
9. Cigarette and e-cigarettes companies will be required to disclose product content to the Ministry of Health (From 06/2020).

## Discussion

The last-minute victory, which overcame massive efforts by the tobacco lobby [7] and years of stagnation in policy-making, occurred after years of intensive advocacy effort, and just prior to the early disbanding of the 20th Knesset. None of the extensions, including Israel’s 2011 National Plan for the Reduction of Smoking and Damage from Smoke [1], included such dramatic changes as does the current law.

### Factors related to success

The reasons for the success were multi-dimensional. Most important to the success was the political will brought to bear by several Members of Knesset (MKs) from a range of political parties. One MK from the Coalition threatened to withhold support for high-priority legislation unless the government backed him on tobacco control regulation. The chair of the Economics Committee promoted the legislation despite strong opposition and lobbying, particularly by the tobacco industry (both local and transnational). Representatives of the vaping industry, newspapers and magazines, and local retail stores were also present during lawmaking and voiced strong opposition. Some MKs agreed to support the legislation due to party obedience, while others across the entire spectrum of political parties became convinced of the urgent necessity for change due to the 8000 annual Israeli deaths due to tobacco [8].

The support of key MKs did not occur in a vacuum. The existence of the FCTC as an international framework [6], and its ratification by the Israeli Knesset [2]; the 2011 governmental passage of a tobacco control plan [1]; and the State Comptroller’s report which criticized governmental and organizational inaction on tobacco control [5], were all important elements. An additional contributing element was the tobacco control coalition which was formed, with the primary immediate goal the passage of the proposed Advertising Bill, which aimed to protect public health, particularly the health of minors,^1^ in the strongest form possible. Avoidance of previous mistakes – including unwillingness to compromise on key issues, particularly the exemption of the print press [2] – was critical. Various individuals and organizations from a wide range of disciplines, including public health and medicine, public policy, public administration, and communications collaborated, some for the first time together, to promote the legislation. Organizations involved were (in alphabetical order): the Association for Progressive Democracy, the Clean Air Society, the Committee to Limit Smoking, Healthy Cities Network, the Israel Association of Health Promotion Professionals, the Israel Association of Public Health Physicians, the Israel Cancer Association, the Israel Council for Smoking Cessation, the Israel Medical Association for Smoking Prevention and Cessation, the Israel Medical Association, Midaat for Informed Health, and Smoke-free Israel. The anomic structure [9] allowed each of the groups to use its particular strengths for the passage of the law. For example, the Society for the Advancement of Democracy focused on dealing with lobbyists, while the Physicians Organizations, the academia, and the Israel Cancer Society worked as advocates for public health. The Association for Smoking Prevention focused on legal aspects, while Smoke-Free Israel dealt with both legal and public policy aspects. Coalition partners from various organizations worked with the media, and were in direct contact with lawmakers throughout the process, by attending Knesset meetings and in private meetings, phone calls, and written communication. Coalition members communicated frequently via social media as well as through more traditional email, phone meetings, and in-person meetings. Joint documents were written and publicized through the local media and at Knesset meetings. Deflecting interference from the tobacco industry and the print press, among others, was critical. Leading journalists kept the public informed and the politicians on their toes with systematic coverage of the issues, as they had done with previous tobacco control efforts [10].

### Deficiencies of the law

The law has clear deficiencies, notably the lack of graphic warnings, the exemption of the print press from the advertising ban, the ability for direct mail (hard-copy materials only, not digital) marketing for people above age 21 who provide advance written consent, the lack of a ban on flavourings, and the lack of a system for enforcement or resources for enforcement. The original draft of the legislation which was part of the 2011 governmental plan for tobacco control [1] included graphic warnings, but these were omitted due to opposition by the Deputy Minister of Health, who opposed graphic warnings on “aesthetic grounds”. The original legislation also included a complete advertising ban, but a condition for governmental support for the bill was an exemption from the advertising ban for the print press. The exclusion of the flavor ban was the result of the lateness of its introduction to the process, and was agreed upon in order to avoid jeopardizing the multi-party agreement on the legislation.

The biggest lacuna in this law is the lack of a clear enforcement mandate and plan. In Israel, enforcement of laws is frequently problematic. In addition, in this particular case, the amendment was not a governmentally-sponsored bill, but a privately-sponsored bill. The original bill, drafted as part of the 2011 governmentally-approved tobacco control plan, included clauses for enforcment [1]. However, that original bill was rescinded by the Ministry of Health in 2014 [2], and when it was re-introduced as a private bill in 2017 the sections on enforcement were deleted.

#### The law and the Richmond Model

This hard-won victory for the health of the Israeli people was much more complex than was the 2011 passage of the National Plan. The passage of that plan was well-explained by the Richmond Model [1], which postulates that a knowledge base (of local conditions and evidence-based policies), a social strategy, and political will are essential elements in prevention policy. The knowledge base for controlling use of combustible tobacco is strong, and includes measures such as tobacco display bans, plain packaging, graphic warnings. However, the knowledge base about the risks and benefits of e-cigarettes and other non-combustible tobacco products is evolving, and at present the professional community has not reached a consensus about how best to regulate them [11]. Likewise, the social strategy which was carefully developed in the context of Healthy Israel 2020 was years out of date, and did not address either heated tobacco products or e-cigarettes [12, 13]. Furthermore, the component of political will was more complicated than previously. The Ministry of Health led the development of the National Plan and its governmental passage. By contrast, the current legislation was initiated and heavily promoted by others, with support from the Ministry of Health. Tobacco industry involvement was extensive. Representatives from national and trans-national tobacco and vaping companies met with Knesset members and actively participated in Knesset meetings [14]. Statements from tobacco industry representatives during Knesset sessions were promptly responded to by representatives of the Association for Progressive Democracy, with reminders of the need to implement Article 5.3 of the FCTC. As in other places, the role of an active coalition was crucial [15].

#### Regulatory changes which occurred in 2019, after the 2018 legislation

Two major regulatory changes occurred in 2019. First, taxation on roll-your-own cigarettes was equalized with taxation on regular cigarettes. This was a major achievement which occurred following extensive activities by coalition partners, and was led by Smoke-Free Israel. Second, there were extensions to existing smoking cessation services. Types of approved behavioral counseling were expanded, dependent on the particular HMO, to include group counselling, telephone counselling, individual intensive behavioral counselling, and/or physician assisted brief behavioral counselling. In addition, the status of nicotine replacement therapy (NRT) was changed so that instead of being considered a second line treatment, to be used only if smokers were medically unable to use varenicline or bupropion, it was also considered a subsidized, first-line medication for smokers using any form of free behavioral counselling provided by their Health Maintenance Organization (HMO).

#### Relationship to activities in other countries

Compared with regulations in other countries, some innovative elements are included, particularly plain packaging for e-cigarettes, warnings on 30% of e-cigarette packaging, and package inserts on all tobacco and smoking products. The combination of plain packaging, package inserts and warnings on 65% of the tobacco products package (with some exemptions for cigars and pipe tobacco) is new. Regulation of heated tobacco products such as Philip Morris’s IQOS under tobacco laws was achieved prior to this legislation, following extensive actions by the tobacco control community [4, 10].

The World Health Organization recognized this progress in May, 2019, when it conferred the World No Tobacco Day Award on one of the organizations involved, Smoke-Free Israel. The letter stated that “recent changes in tobacco control policy in Israel inspire other countries to follow the leadership of Israel and undertake next bold steps in putting health first.” [16]

Despite the extensive progress achieved with this legislation, the Israeli MPOWER score improved only by one point in one measure (Warning labels on tobacco packaging).

Beside the previously mentioned deficiencies in the law, this is also due to the fact that the MPOWER scoring system is limited, and does not include many of the more comprehensive measures advocated by the FCTC, or the newer vaping and heated tobacco products.

#### Beyond research, towards advocacy

For decades, tobacco control leaders have been promoting the importance of going beyond research, and towards advocacy, to reduce the great burden of tobacco in society. Nearly 20 years ago, Simon Chapman noted “a growing recognition of advocacy as a core skill needed in public health practitioners trying to play productive roles in building safer and healthier communities.” [17] Similarly, Judith Mackay’s words of nearly a decade ago ring true: “Tobacco control takes health professionals to very new destinations, away from the traditional curative medical model to mastering the corridors of power, using the media, and political lobbying and advocacy.” [18] This case study is an example of how tobacco control professionals and advocates from many disciplines worked together to advance public health.

## Conclusions

This legislation is an extraordinary leap in tobacco control policy for Israel. Many deficiencies in existing tobacco control regulation were overcome, and some measures even went beyond current international regulations. Strong political leadership, the ratification of the FCTC, the existence of a governmentally-approved tobacco control plan, the State Comptroller’s report of lacunae in tobacco control, and a strong tobacco control coalition contributed to the success. The ad-hoc nature of the coalition allowed for flexibility of each organization to work according to its own principles and methods. For all interested in the health of societies, this is a moment to celebrate. It is now time to: 1) prepare for careful implementation and enforcement of the new legislation; 2) develop a rigorous monitoring system to track population use of tobacco and nicotine products and policy implementation; and 3) plan for further strong measures, which should include increasing the minimum age for tobacco sales to 21 instead of 18, restricting flavors in all tobacco and nicotine products, placing graphic warnings on all tobacco, nicotine, and smoking products, including the print press in the advertising ban, and wise regulation of non-combustible products, in order to achieve a future free of death and disability due to tobacco use. Strong enforcement is crucial for all measures. This case study provides important lessons for up-to-date tobacco control policy, in the age of rapid global changes in the tobacco, vaping and nicotine landscape.

## Data Availability

Data sharing not applicable to this article as no datasets were generated or analyzed during the current study.

## Footnotes

1 “*The purpose of this Law is to protect public health, and particularly the health of minors, by means of establishing prohibitions, restrictions, and obligations concerning smoking products, including the prohibition of advertisements for smoking products and restrictions on actions likely to encourage commencement of use of smoking products or to facilitate their use, as well as by means of providing information for the public, all this due to the serious and fatal damage caused to health pursuant to the use of smoking products;”*
